# Cytokinetic contractile ring structural progression in an early embryo: positioning of scaffolding proteins, recruitment of α-actinin, and effects of myosin II inhibition

**DOI:** 10.3389/fcell.2024.1483345

**Published:** 2024-09-27

**Authors:** John H. Henson, Gabriela Reyes, Nina T. Lo, Karina Herrera, Quenelle W. McKim, Hannah Y. Herzon, Maritriny Galvez-Ceron, Alexandra E. Hershey, Rachael S. Kim, Charles B. Shuster

**Affiliations:** ^1^ Department of Biology, Dickinson College, Carlisle, PA, United States; ^2^ Friday Harbor Laboratories, University of Washington, Friday Harbor, WA, United States; ^3^ Department of Biology, New Mexico State University, Las Cruces, NM, United States

**Keywords:** cytokinesis, contractile ring, myosin II, septin, anillin, actin, sea urchin, α-actinin

## Abstract

Our knowledge of the assembly and dynamics of the cytokinetic contractile ring (CR) in animal cells remains incomplete. We have previously used super-resolution light microscopy and platinum replica electron microscopy to elucidate the ultrastructural organization of the CR in first division sea urchin embryos. To date, our studies indicate that the CR initiates as an equatorial band of clusters containing myosin II, actin, septin and anillin, which then congress over time into patches which coalesce into a linear array characteristic of mature CRs. In the present study, we applied super-resolution interferometric photoactivated localization microscopy to confirm the existence of septin filament-like structures in the developing CR, demonstrate the close associations between septin2, anillin, and myosin II in the CR, as well as to show that septin2 appears consistently submembranous, whereas anillin is more widely distributed in the early CR. We also provide evidence that the major actin cross-linking protein α-actinin only associates with the linearized, late-stage CR and not with the early CR clusters, providing further support to the idea that α-actinin associates with actomyosin structures under tension and can serve as a counterbalance. In addition, we show that inhibition of actomyosin contraction does not stop the assembly of the early CR clusters but does arrest the progression of these structures to the aligned arrays required for functional cytokinesis. Taken together our results reinforce and extend our model for a cluster to patch to linear structural progression of the CR in sea urchin embryos and highlight the evolutionary relationships with cytokinesis in fission yeast.

## Introduction

In contrast to the well-defined process of cytokinesis in fission yeast, fundamental questions remain about the structure and assembly of the cytokinetic contractile ring (CR) in animal cells (Mangione and Gould, 2019; Pollard and O’Shaughnessy, 2019). The rapidly dividing early embryos of sea urchins and other echinoderms have served as the basis for foundational studies on the process and mechanism of cytokinesis, including aspects of cleavage plane determination (Rappaport, 1961), the initial TEM characterization of the actomyosin CR (Schroeder, 1972), the essential role for myosin II in CR contraction (Mabuchi and Okuno, 1977; Kiehart et al., 1982), and the involvement of the G protein RhoA and spindle microtubules in the regulation of cytokinesis positioning and initiation (Mabuchi et al., 1993; Bement et al., 2005; Foe and von Dassow, 2008; Su et al., 2014). Our previous work employing super-resolution light microscopy and platinum replica TEM of first division sea urchin embryos has demonstrated that the CR initiates as a band of clusters of myosin II plus the CR constituent proteins actin, anillin and septin2 which progresses to a linearized actomyosin array consistent with a purse string closure mechanism (Henson et al., 2017; Garno et al., 2021). The aligned actomyosin array we have demonstrated in the late stage sea urchin CR is consistent with super-resolution light microscopy studies of the late stage CR in cultured mammalian cells (Beach et al., 2014; Fenix et al., 2016).

Two CR constituents of particular significance in terms of helping organize CR assembly are the scaffolding proteins anillin and septin with their ability to interact with each other, the membrane, as well as actin and myosin II (Oegema et al., 2000; Straight et al., 2005; Maddox et al., 2007; Piekny and Glotzer, 2008; Bridges and Gladfelter, 2015; Glotzer, 2017; Spiliotis, 2018; Carim et al., 2020). Hickson and colleagues propose that the “anillo-septin” network within the CR can be thought of as another RhoA-activated entity separate from the actomyosin network given the ability of anillin to bind to RhoA-GTP, to recruit septin, and to regulate CR membrane anchorage and constriction (Hickson and O’Farrell, 2008; Carim et al., 2020; Carim and Hickson, 2023). Despite the importance of these scaffolding proteins, their exact architecture within the context of the forming animal cell CR remains unknown. Recently we have employed two super-resolution light microscopy methods - 3D-structured illumination microscopy (3D-SIM, Gustafsson et al., 2008) and stimulated emission depletion microscopy (STED, Hell and Wichmann, 1994) to examine the organization of anillin and septin in the CR of dividing sea urchin embryos (Garno et al., 2021). We have demonstrated their centralized location within myosin II clusters in the early CR, and shown evidence of septin filament-like structures in the later CR. In the present study we have applied the superior 3D resolution of interferometric photoactivated localization microscopy (iPALM, Shtengel et al., 2009) to further investigate the organization of the anillo-septin network scaffold in the development of the first division CR in the sea urchin embryo.

In the present study we have also sought to augment our characterization of the CR by examining the positioning and dynamics of the canonical actin filament crosslinking protein, α-actinin. A number of past studies have shown an association between α-actinin and the CR (Foley and Young, 2014; Leite et al., 2019), including the initial early CR localization work in isolated chick embryo cells (Fujiwara et al., 1978) and sea urchin embryos (Mabuchi et al., 1985). However, the structural organization and timing of the association of α-actinin with the CR remains unclear in animal cells, as does its precise functions. In fission yeast, there is evidence of the importance of the interaction between α-actinin and myosin II in the organization and structural evolution of the CR from nodes to a linearized ring as accomplished by the search, capture, pull, and release mechanism (Wu et al., 2001; Laporte et al., 2012; Lee et al., 2012; Li et al., 2016). In animal cells, CR localization of α-actinin has been shown to be dependent on the F-actin binding and crosslinking domains (Low et al., 2010), and the general assumption is that α-actinin operates as a brake or balance to actomyosin contraction in the CR (Mukhina, et al., 2007; Foley and Young, 2014; Leite et al., 2019), as evidenced by overexpression of α-actinin inhibiting cytokinesis whereas depletion accelerates the cell division process (Mukhina et al., 2007). In the present study, we investigate the hypothesis that α-actinin is not a constituent of the myosin II clusters of the nascent CR of the sea urchin first division embryo, but instead becomes affiliated with the mature CR only during the coalescence and linearization phase due its role in crosslinking elongate actin filaments and the fact that its localization may be evidence of a role as a counterweight to actomyosin contraction (Foley and Young, 2014).

The final aspect of the present study is our effort to dissect out the role of myosin II-based contraction in the context of CR development in the sea urchin embryo CR. A small molecule inhibitor of myosin II ATPase activity, blebbistatin, was discovered via a high throughput small molecule screening approach (Straight et al., 2003), and has been extensively used for the inhibition of smooth muscle and non-muscle myosin II activity to study cell movement and cell shape changes including cytokinesis (Straight et al., 2003; Fenix et al., 2016; Zhang et al., 2017). Despite this long track record of use in research, the utility of blebbistatin is limited by its photosensitivity, toxicity, high autofluorescence, low potency, and poor solubility (Várkuti et al., 2016; Roman et al., 2018). Blebbistatin’s poor solubility has proven particularly problematic in sea water, where the drug forms aggregates that lower the actual effective concentration resulting in highly inconsistent results. More recently, a more soluble, non-toxic, photostable, and non-fluorescent derivative of blebbistatin, para-aminoblebbistatin (PAB), has been synthesized and shown to retain myosin II ATPase inhibitory properties (Várkuti et al., 2016; Roman et al., 2018). In the present study we have used PAB to test the hypothesis that actomyosin-based contraction is dispensable for the initial assembly of the clusters that constitute the nascent CR but essential for the structural progression to the linearized array present in the late CR.

The results of this study extend our previous work characterizing the structural organization and dynamics of the sea urchin embryo CR. We use iPALM super-resolution imaging to confirm the existence of septin filament-like structures in the nascent CR structure, to demonstrate the close associations between septin2, anillin, and myosin II in the CR, as well as to show that septin2 appears submembranous whereas anillin is more widely distributed in the CR. We also provide evidence that the major actin cross-linking protein α-actinin only associates with the linearized, late-stage CR and not with the early CR clusters, providing further support to the idea that α-actinin associates with actomyosin structures under tension and can serve as a counterbalance. In a related finding we show that inhibition of actomyosin contraction does not stop the assembly of the early CR clusters but does arrest the progression of these structures to the aligned arrays required for functional cytokinesis. Taken together our results reinforce and extend our model for a cluster to patch to linear structural progression for the development of the CR in first division sea urchin embryos.

## Materials and methods

### Animals, cell lines, antibodies, drugs, and reagents

Sea urchins of the species *Strongylocentrotus purpuratus* were obtained from Marinus Scientific (Lakewood, CA) or collected from Slip Point, a wave-exposed intertidal site in Clallam Bay, WA, United States (48.26260, −124.2532) under the auspices of a series of Washington Department of Fish and Wildlife-approved scientific collecting permits. Animals collected in WA were maintained in subtidal cages year-round at the University of Washington Friday Harbor Laboratories (FHL, Friday Harbor, WA) or in running natural sea water or closed artificial sea water tanks at 10°C–15°C. Animals kept at FHL were fed *ad libitum* with bullwhip kelp, *Nereocystis luetkeana*, whereas animals in closed artificial sea water tanks were fed dried seaweed from Omega One Seaweed (Blacksburg, VA). LLC-PK1 cells expressing LifeAct-EGFP were obtained courtesy of Dr. Patricia Wadsworth (University of Massachusetts) and cultured according to the methods of Beach et al. (2014).

Primary antibodies used included a rabbit polyclonal antibody raised against sea urchin egg myosin II heavy chain isolated via ATP-based precipitation of actomyosin from *S. purpuratus* egg extracts and electrophoretically purified (Henson et al., 1999), an affinity purified rabbit polyclonal antibody raised against the PH domain of *S. purpuratus* anillin (Garno et al., 2021), a rabbit polyclonal antibody against the C-terminal region of human α-actinin from Santa Cruz Biotechnology (Dallas, TX), a mouse monoclonal antibody against the Ser19 phosphorylated form of myosin II regulatory light chain (P-Myo) from Cell Signaling Technology (Danvers, MA), and a rabbit monoclonal antibody against a peptide from human septin 2 from Abcam, Inc. (Cambridge, MA). Appropriate secondary antibodies conjugated to Alexa Fluor 488, 555, 568, 633, 647, or Oregon Green as well as Alexa Fluor 488, 555, 633, and 647 conjugated phalloidin were obtained from Thermo Fisher Scientific (Waltham, MA). In order to generate secondary antibodies conjugated to the fluorophore Cy3B suitable for iPALM imaging, Cy3b-NHS ester from Amersham Biosciences (Piscataway, NJ) was resuspended to 10 mg/mL in a solvent comprised of a 1:1mixture of dimethyl sulfoxide (DMSO) and dimethyl formamide. Goat anti-mouse IgG and anti-rabbi IgG was resuspended at 1 mg/mL in 0.1 M NaHCO_3_, pH 8.3. Dye and antibody were combined at a molar ratio of 3:1 for an hour at room temperature in the dark. Labeled antibody was separated from free dye using spin columns from an Alexa Fluor Labeling Kit (Thermo Fisher Scientific). Dye labeling was determined using spectrophotometry and working dilutions were determined empirically. Para-aminoblebbistatin (PAB) and Latrunculin A (LatA) were obtained from Cayman Chemical (Ann Arbor, MI). Unless otherwise indicated, the majority of reagents were purchased from either Sigma-Aldrich (St. Louis, MO) or Thermo Fisher Scientific.

### Gamete and coelomocyte collection, fertilization, cleavage cortex isolation, and drug treatments

Sea urchin gametes were collected via intracoelomic injection with 0.5 M KCl, with sperm collected dry and eggs spawned in either natural sea water or MBL artificial sea water (ASW: 423 mM NaCl, 9 mM KCl, 9.27 mM CaCl_2_, 22.94 mM MgCl_2_, 25.5 mM MgSO_4_, 2.14 mM NaHCO_3_, pH 8.0) and subsequently dejellied by multiple washing with ASW. Sea urchin coelomocytes were isolated from the perivisceral fluid of adult animals and maintained in coelomocyte culture media (0.5 M NaCl, 5 mM MgCl_2_, 1 mM EGTA, and 20 mM HEPES, pH 7.2) as described in Smith et al. (2019).

Eggs were fertilized by addition of dilute sperm, the fertilization envelopes removed using 1 M urea (pH 8.0), and then washed into and reared in MBL calcium free sea water (CFSW: MBL ASW minus CaCl_2_ and plus 1 mM EGTA) at 10°C–15°C. Cleavage cortices were generated as described in Henson et al. (2019). In brief, embryos at the appropriate stage of cell division were allowed to quickly settle onto poly-L-lysine (2 mg/mL in 250 mM borate buffer, pH 8.0) coated coverslips and then exposed to fluid shear force from a pipette containing an isotonic cortex isolation buffer (CIB: 0.8 M mannitol, 5 mM MgCl_2_, 10 mM EGTA, 100 mM HEPES, pH 7.4). Isolated cortices were rinsed twice in CIB prior to further processing for fluorescent staining.

For disruption of actin filaments, embryos were treated with either 100 nM LatA or an equivalent dilution of the drug carrier ethanol in CFSW starting at 30 min prior to first division. For myosin II inhibition, embryos 60 min prior to first division were treated with 50 or 100 µM of PAB or the equivalent dilution of the drug carrier DMSO in CFSW. PAB and DMSO were added to CFSW coincident with rapid vortexing in order to ensure dissolution and treated embryos were protected from light exposure given the photosensitivity of PAB. For imaging live embryos treated with PAB, ∼200–300 µL of embryos in CFSW plus PAB were placed on a slide in a well created by nail polish and observed in brightfield or phase contrast microscopy using either a 4X/0.1 NA or a 10X/0.25 NA objective lens on a Nikon (Tokyo, Japan) TS100 microscope. Images were captured using a Photometrics (Tuscon, AZ) QImaging Retiga 2000R cooled CCD camera. For PAB photoinactivation, embryos + PAB were imaged and then exposed to light from four fiber optic light pipes of two Nikon NI-150 high intensity illuminators set at the maximum intensity. After 5–7 min the lights were turned off and the embryos were imaged again to monitor the extent of the reactivation of cytokinesis in the embryos prior to fixation.

### Fixation, fluorescent staining and microscopic imaging and analysis

For widefield or laser scanning confocal fluorescence microscopy, whole embryos, either attached to poly-L-lysine coated coverslips or kept in suspension, were fixed in Millonig’s fixative (0.2 M NaH_2_PO_4_, 0.136 M NaCl, pH 7.0) containing 3.7% formaldehyde for 20 min, washed 3X and then left 2 h to overnight in 0.1% Triton X-100 in phosphate buffered saline (PBST), and then blocked 2 h to overnight in 3% BSA diluted in PBST. Isolated cortices were fixed in 2%–3% formaldehyde in CIB for 5 min followed by blocking in 2% goat serum and 1% BSA in PBS for at least 30 min. Coelomocytes were settled and fixed as described in Smith et al. (2019), and LLC-PK1 cultured cells were fixed in 3.7% formaldehyde in PBS prior to permeabilization in PBST. Immunostaining of all samples was performed with appropriate primary and secondary antibodies diluted in blocking buffer with embryos typically being stained in each antibody step for >1.5 h whereas cortices, coelomocytes, and cultured cells were stained for 45 min in each stage. Fluorescent phalloidin was added to the secondary antibody staining step, and nuclei in embryos and coelomocytes samples were stained in a tertiary step using DAPI in PBST for 10 min. Nuclei in live embryos were fluorescently labeled by incubating cells for 10 min in CFSW containing the membrane permeant DNA dye Hoechst-33342. Fixed samples were typically mounted in nonhardening Vectashield antifade mounting media (Vector Laboratories, Newark, CA) prior to imaging.

For fluorescent staining of isolated cortices for iPALM imaging we followed the methods detailed in Herron et al. (2022). Briefly, cortices were isolated attached to coverslips coated with gold nanorod fiducial markers used for drift correction and calibrations, fixed, quenched using 0.1 M glycine and lysine in PBS, extensively washed in large volumes of PBS, stained, and then mounted in STORM buffer using a second coverslip and this coverslip chamber was sealed with epoxy prior to imaging. iPALM imaging was restricted to secondary antibodies or phalloidin conjugated with either Alexa Flour 647 or Cy3B because of the superior photoactivation properties of these two fluorophores.

Two channel iPALM super-resolution microscopy was performed according to Herron et al. (2022) on a custom-built, noncommercial instrument designed by Hess and colleagues (Shtengel et al., 2009) and housed in the Advanced Imaging Center at the Janelia Research Campus of the Howard Hughes Medical Institute (HHMI-JRC). Samples contained in coverslip chambers were excited with either a 560 nm or a 640 nm laser and emission viewed using opposed, dual Nikon 60X/1.49 NA Apo TIRF objective lenses, with images captured using three iXon3-DU897E EMCCD cameras (Andor Technologies, Abingdon, United Kingdom). iPALM imaging of samples was very low throughput due to the requirement for high staining intensity/density, the slow search process for occasional specimens, and the requirement that tens of thousands of images of a single sample had to be captured over several hours. Images were processed and rendered as 3D tiff stacks using open source PeakSelector software developed at HHMI-JRC. The 3D stacks were rendered using a XY pixel size of 20 nm and a Z step interval of 10 nm and processed to allow for two channel color overlay and alignment, and the fluorescent signatures of the coverslip-associated nanorod fiducial markers were removed.

Conventional widefield epifluorescence microscopy of samples was performed on either a Nikon 80i microscope using either a 40X/0.75 NA Plan Fluorite or 60X/1.4 NA Plan Apo phase contrast objective lens with digital images captured using a Photometrics CoolSnap Cf cooled CCD camera or a Nikon TE2000 microscope using either a 40X/0.95 NA or 60X/1.4 NA Plan Apo DIC lens coupled with a Photometrics QImaging Retiga 2000R cooled CCD camera. Laser scanning confocal microscopy was performed on a Leica (Wetzlar, Germany) SP8 TCS instrument using either a 40X/1.3 NA or a 63X/1.4 NA Plan Apo objective lens.

For all forms of microscopic images, final processing and analysis was performed using Fiji/ImageJ (Bethesda, MD) and/or Imaris Viewer (Oxford Instruments, Abingdon, United Kingdom). Graphs were prepared and statistical analysis carried out using GraphPad Prism 8 (San Diego, CA), with box and whiskers plots generated having the following attributes: all data points are shown, boxes extend from the 25th to the 75th percentile, the horizontal line within the box marks the median, and whiskers extend to the minimum and maximum values. Final figures were prepared using Adobe Photoshop (San Jose, CA).

### Gel sample lysates and immunoblotting

Gel sample lysates of either sea urchin eggs/embryos or LLC-PK1 cells were generated by pelleting cells and then adding hot 2X sample buffer (625 mM Tris-HCl pH 6.8, 25% glycerol, 2% SDS, 0.01% bromophenol blue, 5% β-mercaptoethanol) at four times the volume of packed cells followed by boiling for 5 min. Gel samples were loaded into a 4%–15% Mini-PROTEAN TGX precast protein gel (Bio-Rad, Hercules, CA) and transferred onto nitrocellulose for blotting. Total protein staining of nitrocellulose blots was performed using 0.1% Ponceau S in 5% acetic acid. Blocking and antibody incubation was performed in TBS-Tween supplemented with 3% instant milk. Bound antibody detection was performed for immunoblots using alkaline phosphatase conjugated secondary antibodies and a BCIP + NBT substrate.

## Results

### iPALM imaging of the developing CR confirms the presence of septin filaments and demonstrates the Z axial positioning of septin and anillin

**FIGURE 1:**
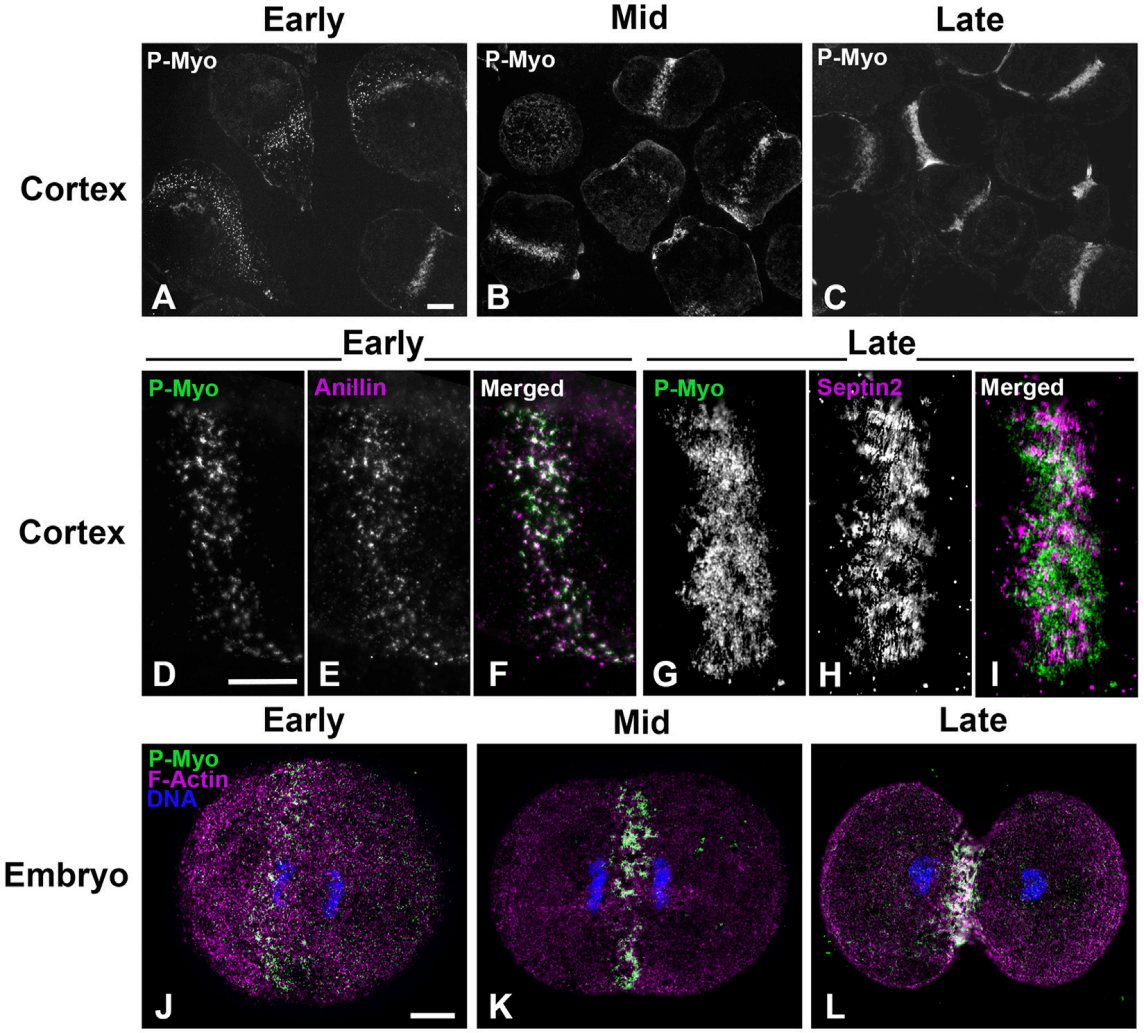
CR structure in isolated cortices and whole embryos evolves from a band of clusters to a dense, linearized array. Early cortices **(A, D–F)** and embryos **(J)** contain a nascent CR consisting of a band of focal clusters of myosin II (P-Myo), anillin, and septin2. This organization congresses into denser patches **(B, K)** and then linear arrays characteristic of the mature CR **(C, G–I, L)**. Scale bars = 10 µm.

Figure 1 shows widefield imaging of isolated cortices and confocal imaging of embryos immunofluorescently (IMF) stained for myosin II (P-Myo) and either septin2 or anillin illustrating the structural progression of the CR from clusters/nodes (Figures 1A, D, E, J) - to patches (Figures 1B, F, G, K) - to linear arrays (Figures 1C, H, I, L). Immunolabeling of whole embryos (Figures 1J–L) fixed and stained in suspension revealed a similar pattern of node to ring structural evolution, suggesting that the observed structures in isolated cortices were not an artifact of the cell adherence to poly-L-lysine coated coverslips.

**FIGURE 2:**
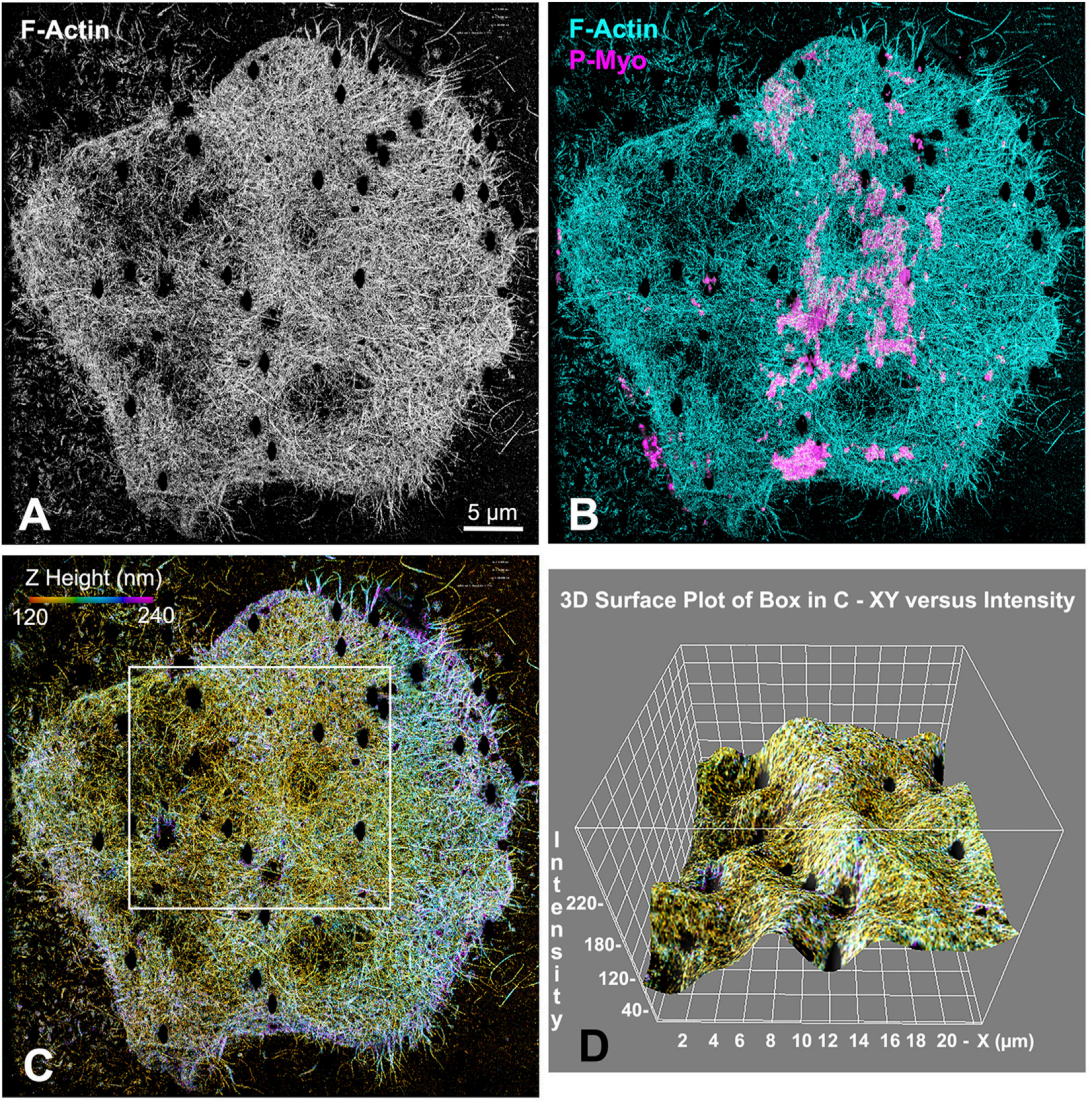
iPALM imaging demonstrates the concentration of F-actin in the CR of an isolated cortex. Maximum intensity projection of iPALM imaging of phalloidin stained F-actin in an isolated cleavage cortex **(A)** shows the CR as an accumulation of actin (cyan) staining running down the middle of the cortex and codistributing with activated myosin II (P-Myo, magenta) localized with near-TIRF imaging **(B)**. Actin filaments in **(C)** are pseudo-colored in accordance with height above the substrate and the surface plot in **(D)** (derived from box in **(C)** shows actin in the mid stripe CR has higher intensity actin labeling and is slightly elevated above the surrounding cortex. Microvillar core bundles of F-actin are most obvious around the edges of the cortex. Note that the black oval holes in the cortex correspond to the positions of gold nanorod fiducial markers that are removed during image processing.

Our earlier work employed 3D-SIM and STED super-resolution microscopy of isolated cortices to characterize the nanostructure of the mature ring as well as the organization of the early clusters (Henson et al., 2017; Garno et al., 2021). However, we needed higher resolution imaging to better visualize the potential existence of CR-associated septin filaments, as well as to define the precise Z axial positioning of septin and anillin in the context of CR organization. Therefore, we employed iPALM super-resolution microscopy developed by Hess and colleagues (Shtengel et al., 2009), which can achieve upwards of 10–20 nm 3D resolution (Shtengel et al., 2009). As a proof of principle, iPALM imaging was performed on phalloidin-labeled actin filaments in isolated first division cortices (Figure 2). These images highlighted the complexity of the actin cortical cytoskeleton which includes the core bundles of microvilli (most evident on the edge of the cortex in Figure 2A), and were similar to the cortical actin cytoskeleton visualized in our previous platinum replica TEM images of cortices (Henson et al., 2017). It also reveals that the CR region, as identified by superposition of near-TIRF imaging of activated myosin II staining (P-Myo, Figure 2B), appears as a ridge of increased relative actin staining intensity as well as relative Z height (Figures 2C, D).

**FIGURE 3:**
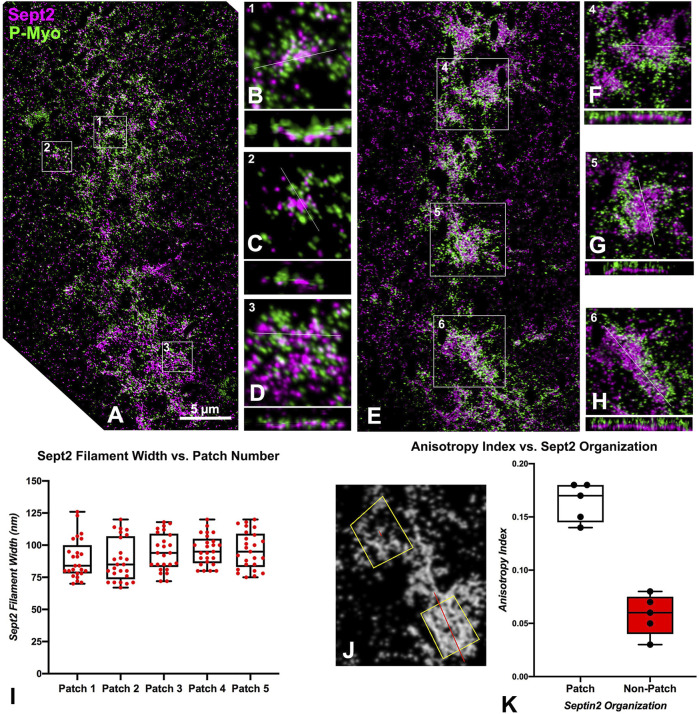
iPALM imaging of septin2 and myosin II in the CR of isolated cortices. **(A)** Maximum intensity projection of an early to mid-stage CR shows a broad band of septin2 (Sept2 - magenta) and myosin II (P-Myo - green) staining. Higher magnification images of the clusters outlined in boxes 1–3 in **(A) (B–D)** show 3 × 3 µm XY and associated 3 × 0.8 µm XZ images (orthogonal reslice plane = line in B-D) in which septin2 and myosin II are closely associated, with septin2 often in the center of clusters in XY, whereas in the Z images the septin2 is associated with the expected submembranous region nearest the coverslip [**(B–D)**, bottom of images]. **(E)** In a mid-stage CR, patches of septin2 and myosin II staining are evident with higher magnification of those outlined in boxes 4–6 **(F–H)** show 7 × 7 µm XY and associated 3 × 0.5 µm XZ images [orthogonal reslice plane = line in **(F–H)**] with filament-like septin2 staining pattern in XY and a clear segregation between submembranous septin2 and deeper myosin II in Z. **(I)** Plot of sept2 filament width in patches shows no significant difference with an average of approximately 93 nm. **(J)** Shows output of FibrilTool analysis of sept2 stained patch (lower yellow ROI box) vs nonpatch (upper yellow ROI box) in which the red line within the ROI box produced by this tool has a length proportional with the anisotropy index and appears at an angle parallel to the axis of orientation of the filamentous elements. Note the red line is significantly longer in the patch vs. the non-patch ROI. **(K)** Plot of measured anisotropy index vs patch and non-patch ROIs shows a significant difference, with anisotropy higher in patch regions.

**FIGURE 4:**
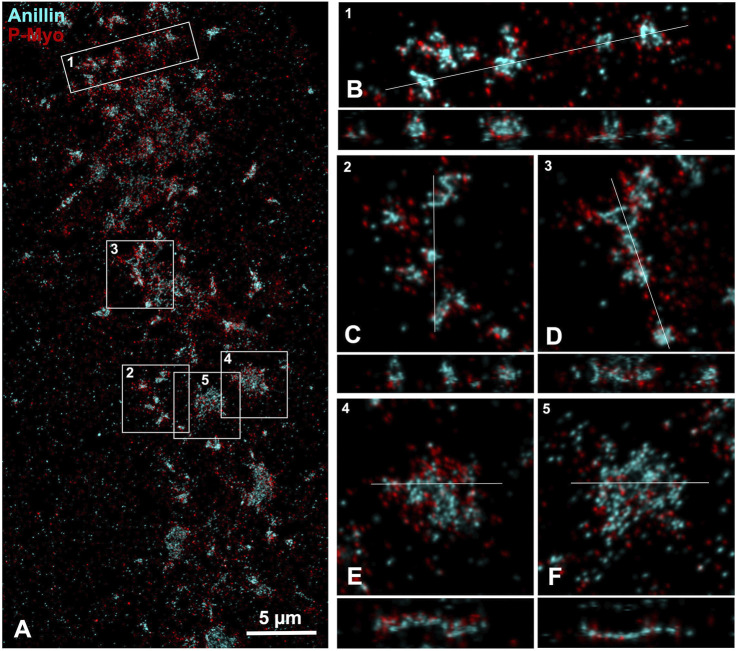
iPALM imaging of anillin and myosin II in the CR of an isolated cortex. **(A)** Maximum intensity projection of an early to mid-stage CR shows a broad band of anillin (cyan) and myosin II (P-Myo - red) staining. Higher magnification images of the clusters outlined in boxes 1–3 **(B–D)** show a 10 × 3 µm XY **(B)** or 5 × 5 µm XY image **(C, D)** plus the associated XZ (Z = 1 µm) axial images [orthogonal reslice plane = line in **(B–F)**]. In these clusters the anillin appears to often reside in the center of the XY images, while in the Z axial images the anillin tends to extend deeper into the cortex and away from the membrane [**(B–D)**, bottom of Z axial images]. Boxes 4 and 5 show patches of closely associated anillin and myosin II staining in XY **(E, F)**, whereas in the Z images the anillin appears aligned along the presumptive submembranous region.

We were able to capture a limited number of iPALM images of CRs in isolated cortices double-labeled for activated myosin II and either septin2 or anillin (three-four cortices per dual labeling from four separate experiments were imaged). This imaging confirmed the close association between activated myosin II and septin2 or anillin in the early clusters (Figures 3A–D, 4A–D), with septin2 and anillin generally located in the cluster center as we showed previously with SIM imaging. In the early-stage clusters (Figures 3A–D) and mid-stage CR patches (Figures 3E–H), Z axial imaging indicated that septin2 was consistently found near the expected plane of the plasma membrane, and between the membrane and staining for myosin II (Figures 3B–D, F–H). Within the mid stage patches the XY images of septin2 staining often appeared as an array of aligned filaments (Figures 3E–H) and image analysis of these patches support this identification in two ways. First, the widths of the septin2-stained filamentous structures are statistically similar between patches (Figure 3I, ANOVA, *p* = 0.16), and they also are similar in value to the widths of contractile stress fiber-associated septin filaments in the human U2OS cell line measured using SIM in the recent study by Martins et al. (2023). Second, the alignment of these filament-like structures is supported by the measurement of a statistically significant increase (t-test, *p* < 0.0001) in anisotropy index in patches versus non-patch organizations (Figures 3J, K). Figure 3J shows the output of this fibrillar analysis on a patch (lower box) and a non-patch septin2 staining organization in which the designated ROI appears as a yellow box and the program analysis appears as a red line within this box, the angle of which represents the orientation of the fibers whereas the length is proportional to the measured anisotropy index (Boudaoud et al., 2014).

iPALM imaging of an anillin and activated myosin II stained cortex shows that early clusters consist of close associations between these proteins in the XY plane (Figure 4), whereas in the *Z*-axis anillin, unlike septin, appears to be spread over a wider distribution within the CR structure (Figures 4B–D). In patch-like accumulations the Z axial imaging shows that anillin appears to be more restricted to a submembranous distribution (Figures 4E, F), similar to what is apparent in the septin2 images (Figures 3F–H). This suggests that anillin localization changes over the course of the structural evolution of the CR which may have relevance for the scaffolding role this protein plays in organizing the CR structure.

### The actin cross-linker α-actinin associates exclusively with the late stage CR

The organization of myosin II, anillin and septins into clusters in the sea urchin CR (Figure 1; Henson et al., 2017; Garno et al., 2021) is reminiscent of the CR precursor nodes in fission yeast (Pollard and O’Shaughnessy, 2019). To further explore how these clusters coalesce into a mature ring, we investigated the importance of the actin crosslinker α-actinin given that in fission yeast CR development*,* proper node condensation depends on the actin crosslinking proteins α-actinin and fimbrin (Laporte et al., 2012; Li et al., 2016). We were interested in testing the hypothesis that α-actinin only associates with the late-stage sea urchin embryo CR that is undergoing actomyosin-based contraction. Sea urchin egg α-actinin has a molecular mass of ∼95 kDa, resembles mammalian α-actinin in rotary shadow TEM, cross-links actin filaments into large bundles, and localizes to the cleavage furrow when fluorescent conjugates are microinjected into fertilized eggs (Mabuchi et al., 1985). Immunoblotting with a commercial rabbit polyclonal antibody specific for the conserved carboxy terminus region of human α-actinin showed the expected immunoreactive band at ∼100 kDa in lysates of cultured mammalian LLC-PK1 cells as well as a similar molecular weight band in *S. purpuratus* sea urchin eggs and first division embryos (Supplementary Figure S1A). Immunolabeling of porcine LLC-PK1 cells with the anti-human α-actinin gives the expected staining of cell margins, the CR in dividing cells, and actomyosin stress fibers (Supplementary Figures S1B–SE) – in which the staining is a characteristic pattern of periodic foci (Katoh et al., 1998). Staining of isolated coelomocytes from adult *S. purpuratus* sea urchins shows that the anti-human α-actinin also codistributes with actin and myosin II and periodically stains actomyosin stress fibers in the cytoskeleton of these somatic cells (Supplementary Figures S1E–SH).

**FIGURE 5:**
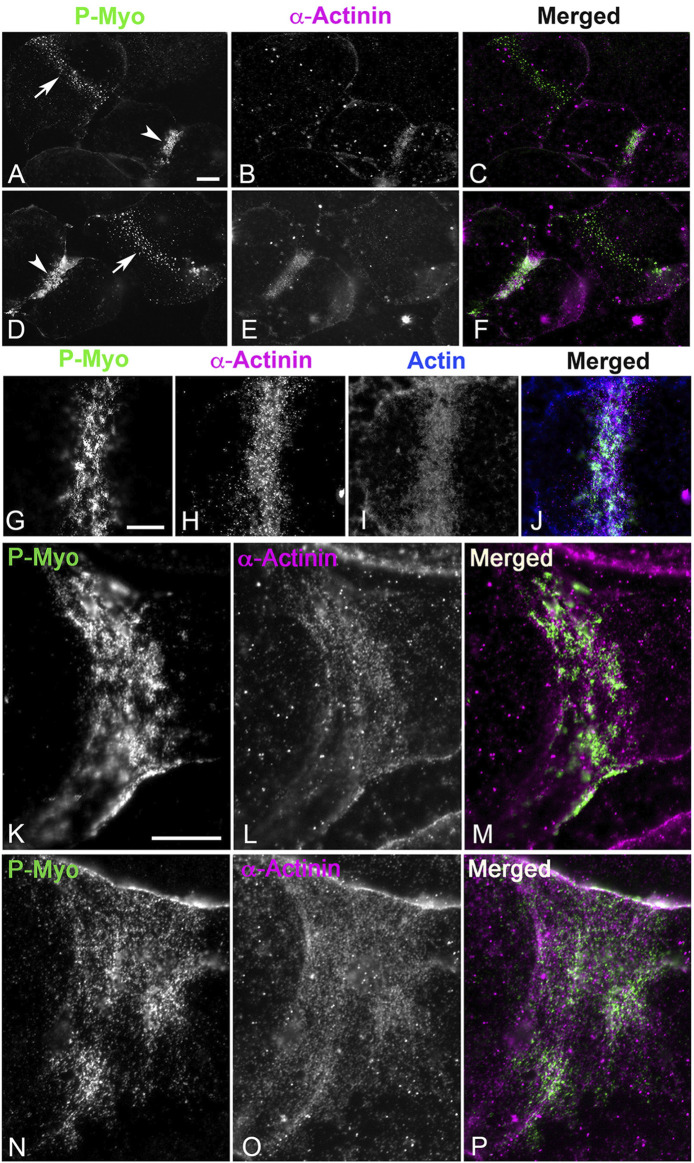
In isolated cortices ⍺-actinin localizes to late - but not early - CRs where it codistributes with linear actomyosin arrays. In cortices stained for activated myosin II (P-Myo) and alpha-actinin **(A–F)**, early stage CRs (arrows in **(A,D)**) containing myosin II clusters do not contain α-actinin, but late stage CRs (arrowheads in **(A,D)**) do concentrate α-actinin, along with actin and myosin II **(G–J)**. In select late stage cortices in which the CR is stretched during isolation, the characteristic discontinuous punctate ⍺-actinin staining of the CR linear actomyosin structure is particularly evident **(K–P)** reminiscent of ⍺-actinin staining of stress fibers in PK1 cells and coelomocytes (Supplementary Figure S1). Bar = 10 μm.

In cortices isolated from first division sea urchin embryos, α-actinin does not stain the myosin II clusters, or patches present in early to mid-stage CRs (Figures 5A–F, arrows) but does associate with the more aligned myosin II linear arrays of later stage CRs (Figures 5A–F, arrowheads). In these later stage CRs, the concentration of α-actinin labeling is associated with a concentration of F-actin staining (Figures 5G–J) suggesting a role in the bundling of CR-associated actin filaments and/or serving as a mechanosensor for actomyosin tension. In select cortices the structure of the late stage, linearized CR is stretched during the isolation process which leads to a significant broadening of its structure (Figures 5K–P). In these cortices, the linear periodic pattern of α-actinin staining of the actomyosin cytoskeleton is particularly clear (Figures 5K–P) and reminiscent of the staining pattern seen in stress fibers of coelomocytes and LLC-PK1 cells (Supplementary Figures S1B–SH). We have shown previously in platinum replica TEM images that the architecture of the actomyosin cytoskeleton is similar in the stress fibers of LLC-PK1 cells and the late-stage CR of dividing sea urchin embryos (Henson et al., 2017).

**FIGURE 6:**
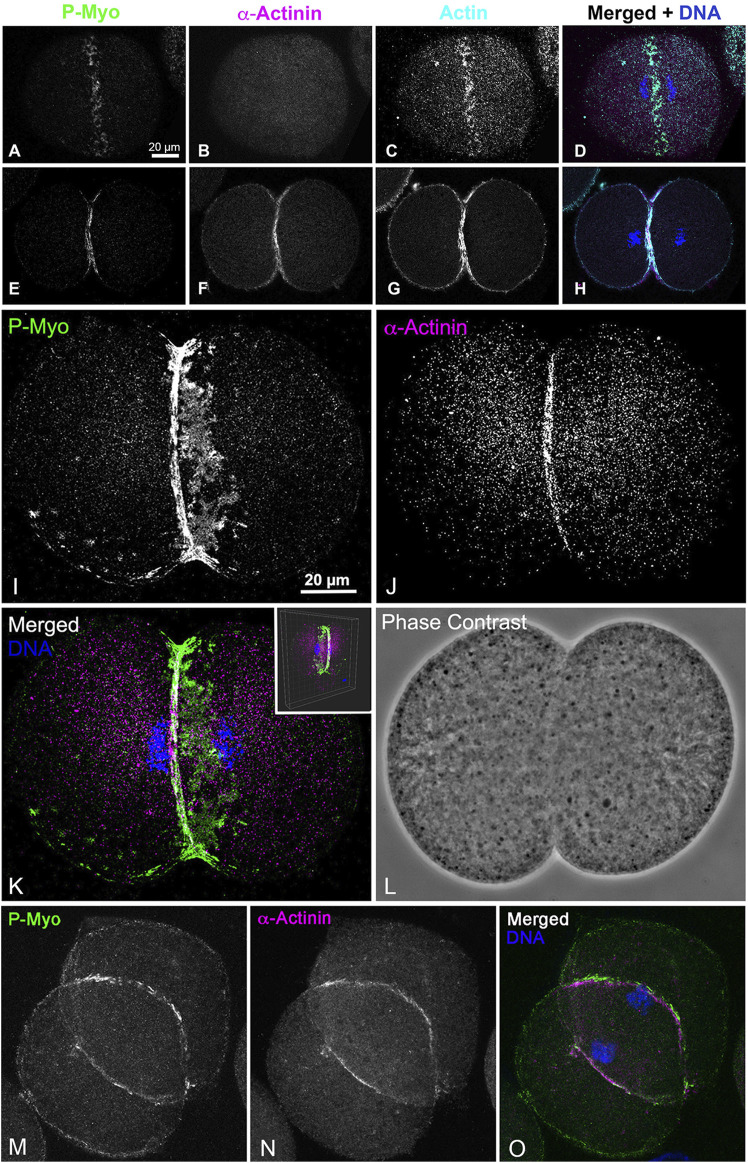
In whole embryos ⍺-actinin also localizes exclusively to late stage, linearized CRs. Early stage CRs in embryos contain activated myosin II (P-Myo) and F-actin clusters but no ⍺-actinin **(A–D)**, whereas later stage CRs stain for all three of these CR constituent proteins **(E–H)**. Late and early CR organizations can exist in the same embryo. A confocal through focus projection **(I–L)** shows a cluster-based CR on one side of an embryo with a linearized CR on the other. Note ⍺-actinin stains only the linearized structure and not the clusters, whereas activated myosin II localizes to both **(I–K)**. Inset in K shows a 3D reconstruction of the merged image. A through focus projection of an embryo with a late stage and fully linearized CR demonstrates staining for activated myosin II and ⍺-actinin throughout **(M–O)**.

The lack of association of α-actinin with the early CR clusters is also observed in whole embryos fixed and stained in suspension (Figures 6A–H). In these embryos, α-actinin only localizes to the mature CR in embryos undergoing furrowing (Figures 6E–H) and not in the myosin II clusters present in unconstricted, late anaphase embryos (Figures 6A–D). Settling embryos onto poly-L-lysine-coated coverslips can slow CR formation on the attached side of the embryo relative to the unattached side, and in these embryos, the α-actinin still only associates with the linearized late-stage CR structure and not with the CR clusters on the attached side (Figures 6I–L). In these same experiments, complete linearized CRs with associated myosin II and α-actinin ring-staining are present in dividing embryos in which the region of the developing CR is not attached to the coverslip (Figures 6M–O).

### Cluster congression requires myosin II activity

**FIGURE 7:**
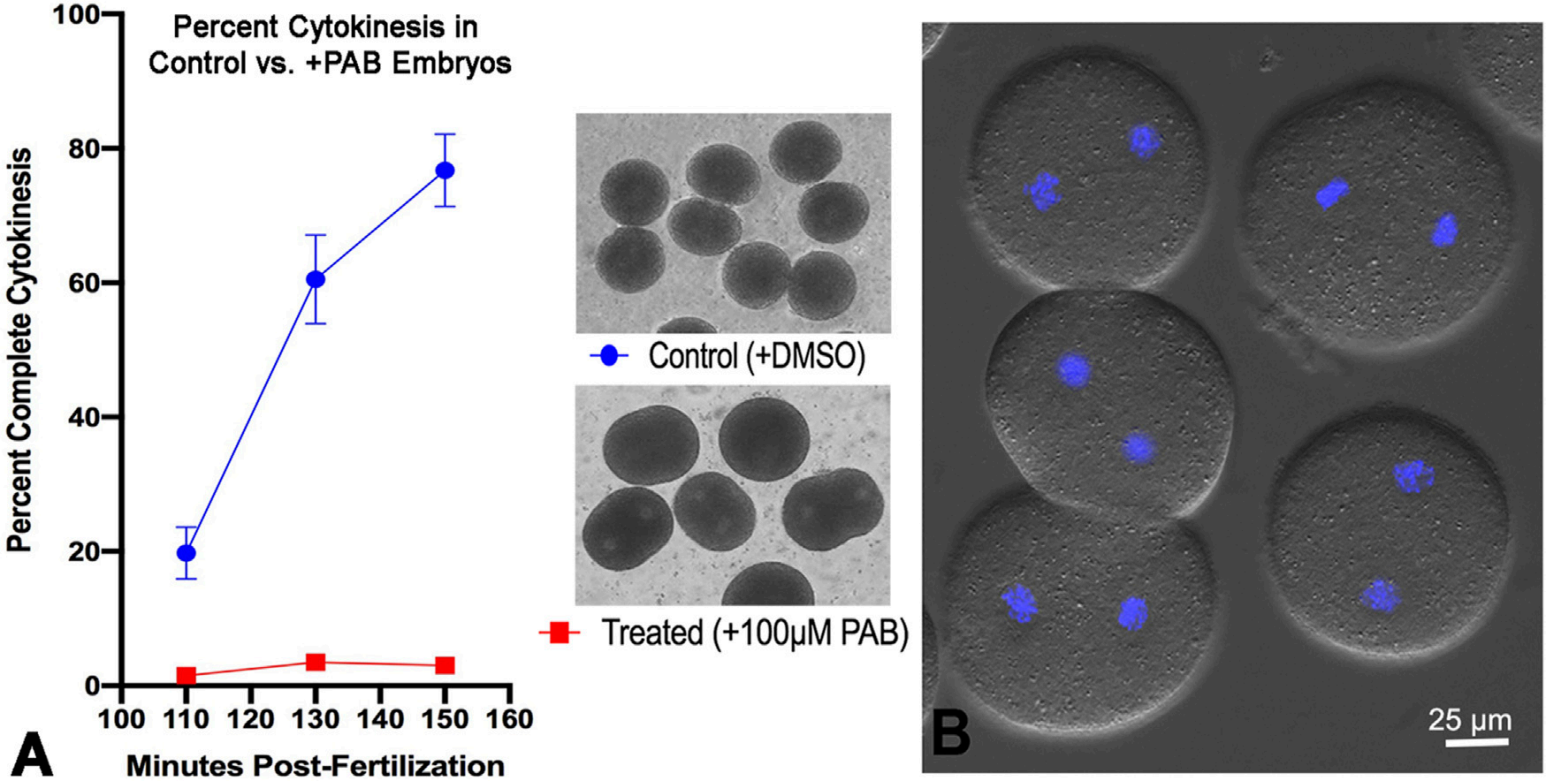
PAB inhibits cytokinesis but not karyokinesis in embryos. A graph of percent completed cytokinesis **(A)** shows the inhibitory impact of 100 µM PAB treatment (red line) relative to DMSO treated controls (blue line). The brightfield image insets in panel A show live control (top) and +PAB (bottom) embryos 140 min post-fertilization in which the controls have divided completely and the +PAB are exhibiting elongation, flattening, slight constrictions, and/or the presence of two nuclei. In DIC plus fluorescence images of live + PAB embryos 160 min after fertilization **(B)** staining with Hoechst-33342 (blue) shows that these embryos are binucleate, confirming that karyokinesis takes place in the absence of cytokinesis.

Tracking cytokinesis completion in first cell cycle *S. purpuratus* embryos clearly shows that treatment with 100 µM of the myosin II inhibitor PAB prevents cytokinesis, although embryos can exhibit anaphase B elongation along with flattening in the equatorial region, typically in a time frame that is 20–30 min later than cytokinesis in the control embryos (Figure 7). The uninhibited progression of karyokinesis means that the PAB treated embryos eventually become binucleate (Figure 7B), and that they continue with additional nuclear divisions that are delayed relative to the timing of cell divisions in control embryos. Further support of the specificity of PAB on the process of actomyosin-mediated cytokinesis is provided by additional experiments showing that PAB displays a dose-dependent impact on cytokinesis inhibition (Supplementary Figure S2). Embryos treated with 50 µM PAB develop significant furrowing relative to the 100 µM treatment, although this lower dose treatment still inhibits full cytokinesis in the majority of embryos, with many embryos becoming binucleate (Supplementary Figure S2).

As an additional functional test of the specificity of PAB as an inhibitor of myosin II contraction in sea urchin cells, we turned to the actomyosin-dependent process of centripetal flow in sea urchin coelomocytes. We have previously shown that discoidal coelomocytes settled on glass exhibit an extensive type of actin-based centripetal flow in which movement at the cell edge is driven by Arp2/3-dependent actin polymerization, whereas movement within the cell center is based on myosin II contraction as assayed by treatment with myosin II light chain kinase and general phosphatase inhibitors (Henson et al., 1999; Henson et al., 2003). Therefore, treatment of discoidal coelomocytes with PAB should arrest central but not peripheral centripetal flow and disrupt the central actomyosin structure. This is the precise result we obtained when treating these cells as demonstrated by live cell phase contrast microscopy and actin and myosin II fluorescent staining (Supplementary Figure S3).

**FIGURE 8:**
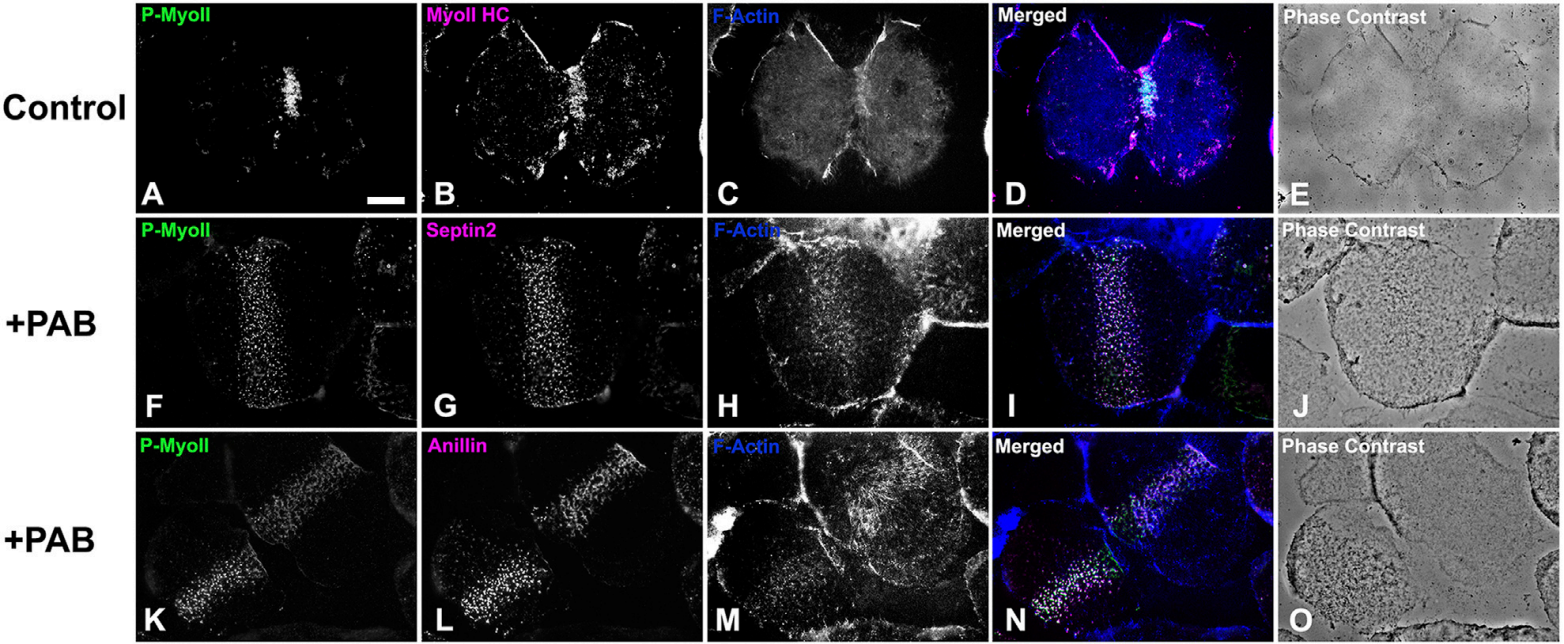
Cortices isolated from PAB treated embryos contain a band of early CR-like myosin II clusters. Control cortices **(A–E)** show contracted CRs containing activated phospho-myosin II RLC [P-MyoII, **(A)**] along with myosin II heavy chain [MyoII HC, **(B)**], and F-actin **(C)**. In contrast similar stage +100 µM PAB cortices **(F–O)** display broad bands of early CR-like myosin II **(F, K)** clusters which also contain septin2 **(G)**, anillin **(L)**, and F-actin **(H, M)**. Linearized late stage CR structures are not present in +PAB cortices. Bar = 10 µm.

**FIGURE 9:**
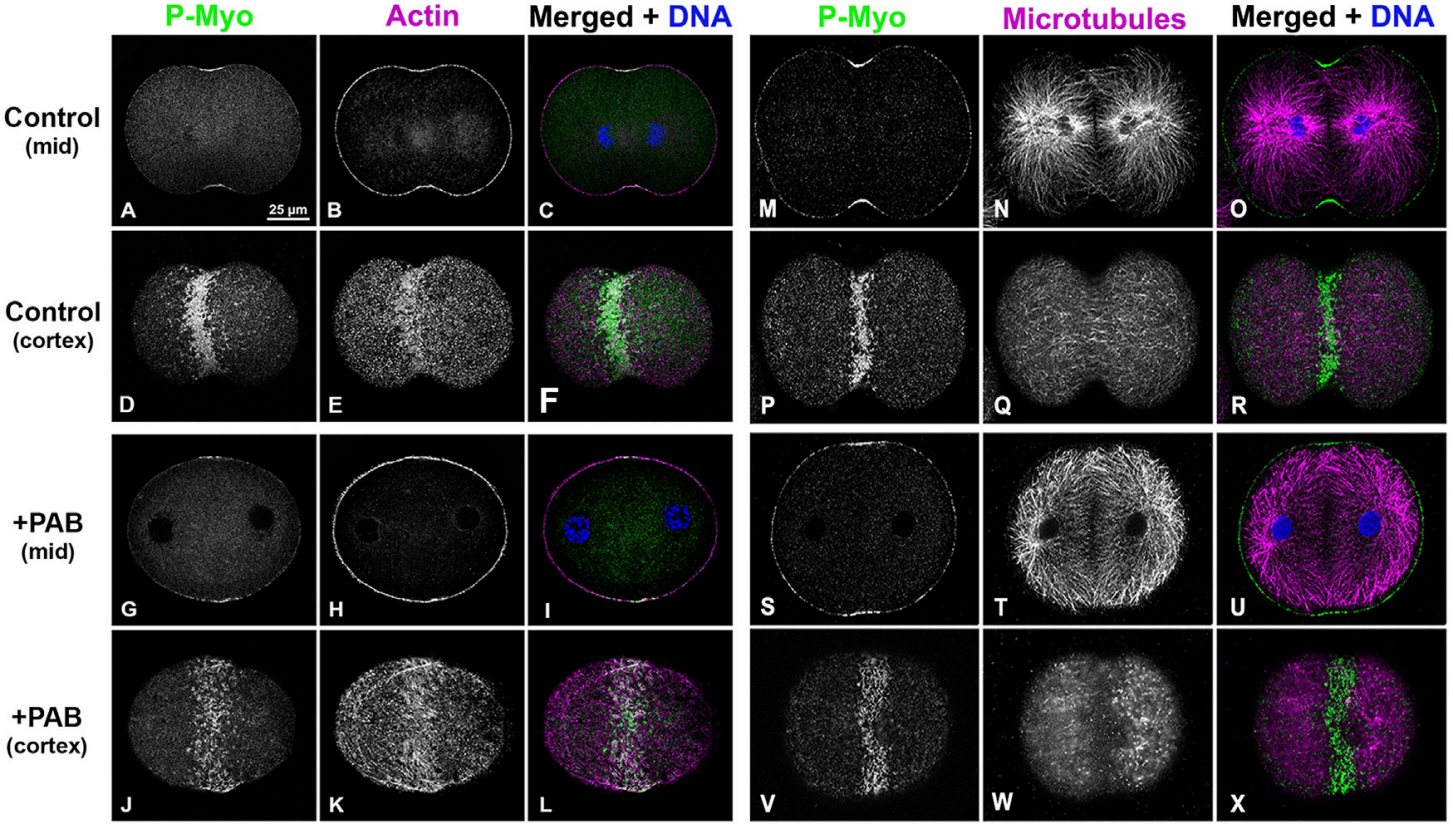
PAB treated embryos contain early CR-like bands of myosin II clusters along with control-like arrays of microtubules. Confocal images of the mid and cortical Z slices of control embryos **(A–F, M–R)** contain constricted CRs with dense assemblages of activated myosin II (P-Myo, A, D, M, P) and F-actin **(B, E)**, along with associated microtubules **(N, Q)**. +100 µM PAB embryos **(G–L, S–X)** contain an early CR-like band of myosin II clusters **(G, J, S, V)** with associated actin **(H, K)** and microtubules **(T, W)**, however linear arrays of CR constituents are not present in these PAB-treated embryos. Furthermore, microtubule organization does not appear significantly disrupted by PAB treatment. Control embryos 130 min post-fertilization and +PAB embryos 140 min post-fertilization.

In order to examine the impact of PAB inhibition of myosin II contraction on cluster aggregation and ring organization, cortices (Figure 8) and whole embryos (Figure 9) derived from PAB and DMSO treated embryos were probed for CR constituents. Cortices isolated from +PAB embryos 140–160 min post-fertilization showed early CR-like structures with broad bands of myosin II nodes which also contained septin2, anillin, and actin (Figure 8). The linearized arrays of CR proteins characteristic of late-stage dividing cortices were not observed in the PAB treated cortices (Figure 8). Similarly, in whole embryos stained in suspension, wide bands of myosin II and actin clusters were present in the often elongated embryos following PAB treatment in the 140–160 min post-fertilization time frame (Figures 9A–L). These PAB treated embryos underwent microtubule-mediated karyokinesis with embryos displaying an early CR-structure and a dense array of microtubules resembling that seen in control telophase embryos (Figures 9M–X). This is important to demonstrate given that one possible off target effect of PAB might be an impact on the cytokinesis-essential interaction of astral microtubules with the equatorial cortex (Su et al., 2014), however the staining suggests that this was not the case. Finally, as a test of the requirement for any actomyosin interaction in the formation of the early CR clusters, we treated developing embryos simultaneously with 100 µM PAB to inhibit myosin II and 100 nM Latrunculin A to eliminate actin filaments prior to first division. Staining of these embryos revealed that the myosin II clusters of the early CR still assembled and microtubule arrays still accomplished karyokinesis despite the absence of actin filaments and myosin II function (Supplementary Figure S4).

The combination of the results of the PAB treatment experiments indicate that inhibition of myosin II contraction does not interfere with the initial assembly of the early CR myosin II clusters, however it does prohibit the coalescence of these structures into a linearized array. In order to test whether the band of myosin II clusters present in PAB treated embryos was capable of transforming into a functional CR, we photoinactivated PAB in treated embryos 140–160 min post fertilization using high intensity white light from fiber optic illuminators (Supplementary Figure S5). Immediately upon photoinactivation embryos commenced cytokinesis-like constriction, including complete abscission (Supplementary Figure S5), suggesting that the nascent CR cluster structure in PAB treated embryos can serve as a template for a functional CR. Immunofluorescent staining shows that embryos undergoing cytokinesis following PAB photoinactivation have the expected concentration of myosin II and actin in the CR along with a microtubule array and decondensed nuclei that are characteristic of PAB treated binucleated embryos that have undergone karyokinesis (Supplementary Figure S5).

## Discussion

Despite clear evidence of the importance of the scaffolding proteins anillin and septin in helping structure the CR in multiple cell types, the precise nature of their structural organization in the context of the CR remains enigmatic outside of the studies performed in yeast (Mangione and Gould, 2019; Carim et al., 2020). In addition, the evidence for the presence of discernible septin filaments in animal cells has been limited. This includes work using SIM to show short septin filaments in the lamellar arcs of migrating cultured mammalian cells (Dolat et al., 2014), PALM/STORM imaging of apparent septin bundles in stress fibers in cultured mammalian cells (Vissa et al., 2019), and our previous SIM and STED imaging suggesting septin filament-like structures in the sea urchin CR (Garno et al., 2021). A recent groundbreaking study by Martins and colleagues (2023) used a split-GFP complementation assay combined with SIM microscopy to demonstrate that octamer-based septin filaments are associated with contractile stress fibers in cultured human cells and appear to mediate actin-membrane anchoring (Martins et al., 2023). In our present study, we employed the superior 3D super-resolution of iPALM imaging to show that septin2 in the developing CR (Figure 3) exists as patches of myosin II-adjacent and submembranous filaments arranged as dense, aligned assemblages similar to the septin filament arrays imaged by TEM associated with membranes *in vitro* (Szuba et al., 2021), and in the cortex of budding yeast (Bertin et al., 2010). The width measurements of these septin filaments are in general agreement with those reported by Martins et al. (2023), and the occasional periodicity of the staining pattern may be due to the distribution of the septin2 subunits within the heterooligomeric structure of the filament. The iPALM Z axial images show septin in close association with the membrane (Figure 3), which is consistent with the membrane adjacent localization of septin seen in fPALM imaging of the fission yeast CR (McDonald et al., 2017). In addition, the presence of these septin filament-like structures on coalesced patches and not the initiating clusters suggests that these filaments associate with actomyosin contractile structures, given that PAB-based myosin II inhibition clearly inhibits the cluster to patch progression (Figures 8, 9). This result is also consistent with evidence from mammalian cells showing that septin filaments associate with contractile and not non-contractile stress fibers (Martins et al., 2023).

Our iPALM images of anillin indicate that this protein extends further away from the membrane and deeper into the adjacent cortex compared with septin (Figures 4B–F). Anillin has been described as the most multifuctional scaffold for the CR given its ability to interact with actin, myosin II, septin, phospholipids, and RhoA, and it has been shown to drive the recruitment of septin to the CR membrane (Sun et al., 2015; Carim et al., 2020; Carim and Hickson, 2023). Therefore, it is difficult to reconcile our iPALM images of more diffuse anillin localization with the role that this protein appears to play in binding septin to the membrane. One explanation might be that models of anillin’s placement in the CR suggest an elongated overall structure which reaches from the membrane and RhoA binding associated with the PH and C2 domains to the actomyosin binding N-terminal domain (Sun et al., 2015; Carim and Hickson, 2023). Our iPALM images do show that anillin in later stage patches appears to be more restricted to a submembranous localization than in the less organized clusters (Figure 4), suggesting that anillin placement within the developing CR organization changes over time, perhaps coincident with the onset of actomyosin contraction. Note that fPALM imaging in the mature CR in fission yeast localizes the yeast anillin analogue Mid1 to the membrane adjacent with septin (McDonald et al., 2017).

In the early sea urchin embryo, α-actinin associates only with the late stage, linearized CR and not with the clusters and patches characteristic of the early CR (Figures 5, 6). This localization pattern could be observed within a single embryo possessing both early and late CR structures (Figure 6), suggesting that α-actinin only associates with the CR once the actomyosin organization supports contraction. The relatively late localization of α-actinin is also consistent with previous results showing that Latrunculin-based disruption of actin in embryos does not prevent formation of myosin II clusters (Foe and von Dassow, 2008), but does stop their structural progression to a linear array (Henson et al., 2017; Garno et al., 2021). The potential mechanosensory activity of α-actinin (Luo et al., 2013; Schiffhauer et al., 2016) may also explain the late association with the CR, and provide further support to the notion that α-actinin may operate as a counterweight to actomyosin tension in the CR (Mukhina et al., 2007; Foley and Young, 2014).

The PAB-based myosin II cytokinesis inhibition results reported here highlight the ability of the early CR myosin II clusters to form in the presence of the inhibition of myosin II contraction (Figures 7, 8) and are consistent with earlier work using its parent drug blebbistatin which showed cytokinesis inhibition in HeLa cells and that myosin II was present in an unconstrictred CR-like organization/concentration in treated cells (Straight et al., 2003; Zhou and Wang, 2008; Fenix et al., 2016; Spira et al., 2017). Straight et al. (2003) also showed that blebbistatin did not significantly alter microtubule arrays in dividing HeLa cells which is similar to what we have observed with PAB treatment in sea urchin embryos (Figure 9; Supplementary Figure S4). Our confidence in the specificity, efficacy, as well as low toxicity of PAB in our sea urchin experimental system is bolstered by the following: the increased solubility and photostability of PAB generated far more consistent cytokinesis inhibition (Figure 7) than that seen in our previous experiments with the parent drug blebbistatin; the dose-dependence of the extent of PAB’s inhibition of cytokinesis (Supplementary Figure S2), the ability of PAB treated embryos to complete cytokinesis when the drug was photoinactivated via high intensity light exposure (Supplementary Figure S5); and the ability of PAB to inhibit the myosin II-based aspect of centripetal flow in sea urchin coelomocytes (Supplementary Figure S3).

In terms of the mechanisms underlying the prevention of cytokinesis by PAB, blebbistatin’s inhibition of the myosin II contraction involves its arrest in a low actin affinity state that inhibits the post-ATP hydrolysis release of P_i_ (Kovacs et al., 2004; Allingham et al., 2005; Zhang et al., 2017; Roman et al., 2018). Therefore, PAB treatment would be expected to interfere both with myosin II-based contraction as well as potential myosin II-based actin crosslinking. A recent study in *C. elegans* embryos expressing myosin II motor-impaired mutants has concluded that myosin II motor activity, and not just actin cross-linking, is required for cytokinesis, at least in this system (Osório et al., 2019). Note that our results showing that early CR myosin II clusters can assemble in embryos treated with both LatA and PAB (Supplementary Figure S4), and therefore devoid of actin filaments and myosin II contraction, indicate that actomyosin interactions in general are not necessary for early CR construction. In addition, our demonstration that PAB treatment displays a dose dependent impact on cytokinesis inhibition (Supplementary Figure S2) is in agreement with previous work examining the dose dependence of blebbistatin treatment on cytokinesis and the associated myosin II minifilament expansion and concatenation into the mature CR of HeLa cells (Fenix et al., 2016). Our earlier work has shown that myosin II minifilaments in the later stage sea urchin CR form aligned/concatenated arrays (Henson et al., 2017) and PAB-based myosin II inhibition appears to prevent this reorganization. Note that an earlier study using blebbistatin treatment of sea urchin embryos has suggested that this drug does not block the process of myosin II bipolar minifilament assembly (Moorhouse et al., 2015).

In conclusion, the results of this present study reinforce and extend our working model for a cluster to patch to linear structural progression of the CR in early sea urchin embryos (Figure 1; Henson et al., 2017; Garno et al., 2021). This model posits that the CR is initiated as focused clusters that are formed from the interaction of the scaffolding proteins anillin and septin with myosin II that are independent of the presence of actin filaments and/or actomyosin contraction (Figures 7–9; Supplementary Figure S4). Then the onset of actomyosin contraction drives the coalescence of these clusters into patches containing a reorganized distribution of anillin (Figure 4) along with septin filamentous structures (Figure 3) that may be critical for CR actin filament-membrane anchoring (Martins et al., 2023). As actomyosin contraction progresses the final stage is the linearization and narrowing of the actomyosin array coincident with the association of the actin filament crosslinking protein α-actinin (Figures 5, 6) which may serve as an important regulatory counterweight to contractile tension during CR closure. In addition, a number of our findings underscore the evolutionary connections between CR assembly in early sea urchin embryos and fission yeast, including the parallels between sea urchin clusters and yeast nodes, roles for the actin filament crosslinker α-actinin, and the involvement of septin filaments.

Numerous significant questions remain about building the CR, including what is specifying the template for the assembly of the CR-initiating myosin II clusters. Previous work has suggested that it might correspond to distribution of PI(4,5)P_2_ and/or RhoA-GTP which would serve as a anchoring site for the master scaffolder anillin (Sun et al., 2015). This could in turn be related to regions of the membrane impacted by the RhoA activating complex centralspindlin moving out along equatorial-targeting astral microtubules (Vale et al., 2009; Argiros et al., 2012; White and Glotzer, 2012). We are in the process of investigating both of these related hypotheses.

## Data Availability

The original contributions presented in the study are included in the article/Supplementary Material, further inquiries can be directed to the corresponding author.
