# Supplementary material for: Cytokinetic contractile ring structural progression in an early embryo: positioning of scaffolding proteins, recruitment of α-actinin, and effects of myosin II inhibition
Source: Front Cell Dev Biol. 2024 Sep 27;12:1483345. doi: 10.3389/fcell.2024.1483345 (PMC11467475; doi:10.3389/fcell.2024.1483345)
Supplement: Supplementary file 6 [file Image1.PDF]

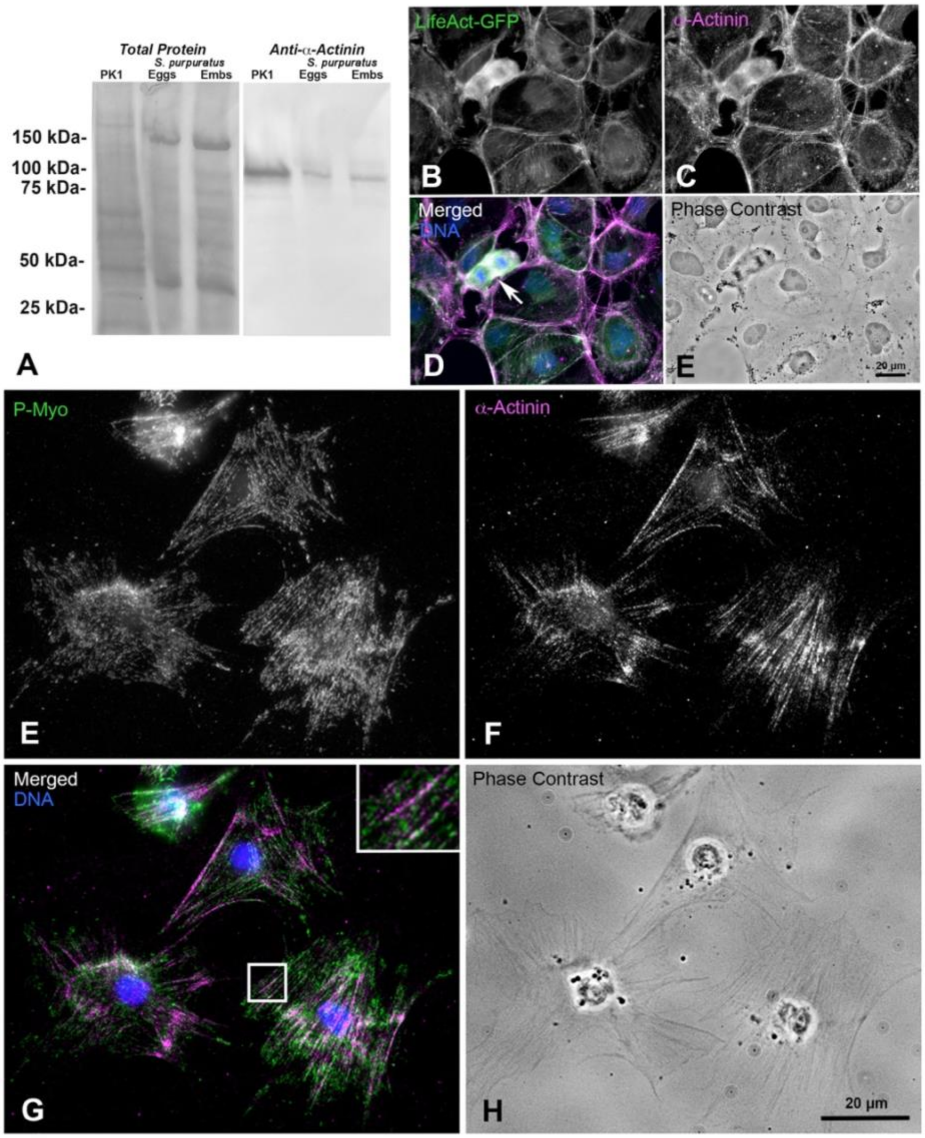

Supplementary Figure S1: An anti-human α-actinin antibody provides characteristic labeling in mammalian cultured cells and sea urchin coelomocytes. Immunoblot of anti-human α-actinin against lysates of mammalian LLC-PK1 cells, sea urchin eggs, and first division sea urchin embryos shows immunoreactive bands of the expected 100 kD molecular weight in each sample. Total protein labeling of this same blot appears on the left with molecular weight markers (A). Using this same anti-α-actinin (magenta) to stain PK1 cells (B-E) shows the expected labeling of actin (green) structures (labeled with LifeAct-EGFP) such as stress fibers, cell margins, and the CR in dividing cells (arrow in D). Staining of sea urchin coelomocytes (E-H) with this anti-α-actinin (magenta) shows clear labeling of stress fiber-like actin bundles which closely associate with activated myosin II (P-Myo, green) staining. The higher magnification inset from the box in G shows the characteristic periodic labeling of these structures with both α-actinin and myosin II.

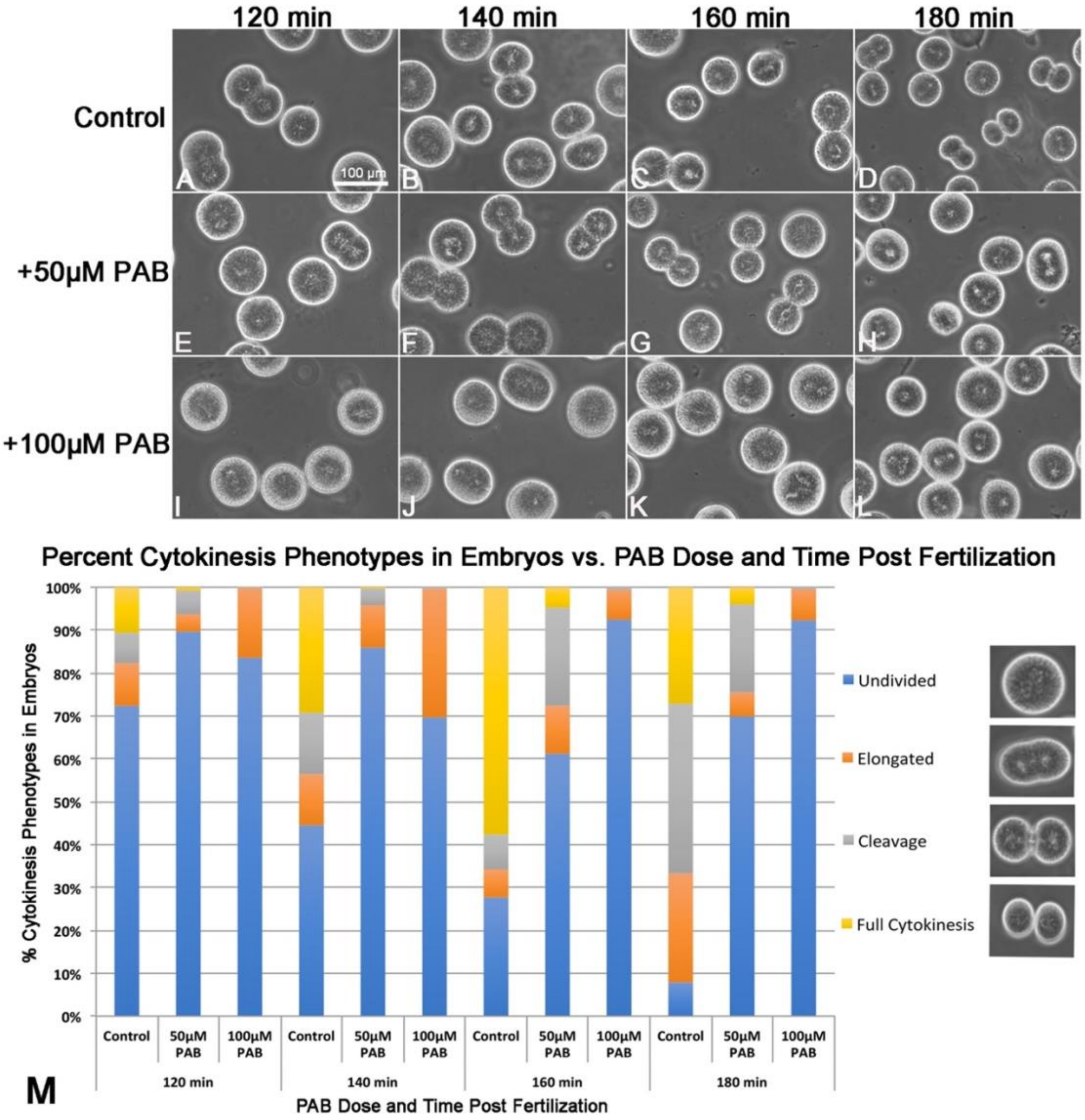

Supplementary Figure S2: PAB has a dose dependent impact on cytokinesis in embryos. Comparing cytokinesis phenotypes (undivided, elongated, cleavage, and full cytokinesis – see images associated with key in graph in M) in embryos fixed at 4 times following fertilization (120, 140, 160 and 180 min) shows major differences in control (A-D), 50 µM (E-H), and 100 µM (I-L) PAB-treated embryos (*note that all embryos were cultured in CFSW and therefore blastomeres dissociate after cytokinesis*). Control embryos (A-D, M) start undergoing their first division at the 120 min time point and progressively accumulate more full cytokinesis embryos up till the 160 min time point, whereas by the 180 min timepoint they begin to undergo the 2-4 cell division (as seen in the smaller blastomeres dividing in panel D). In contrast, +50 µM embryos (E-H, M) show moderate levels of elongation, cleavage, and full cytokinesis phenotypes through F + 180 min, although they can undergo significant cleavage furrowing (as seen in some embryos in F, G). Embryos treated with 100 µM PAB (I-L, M) display only elongation, with minimal cleavage furrowing and no full cytokinesis. Binucleated embryos are present by the 140-160 min timepoints (J, K), and this is also seen by F + 180 min in the +50 µM PAB embryos (H).

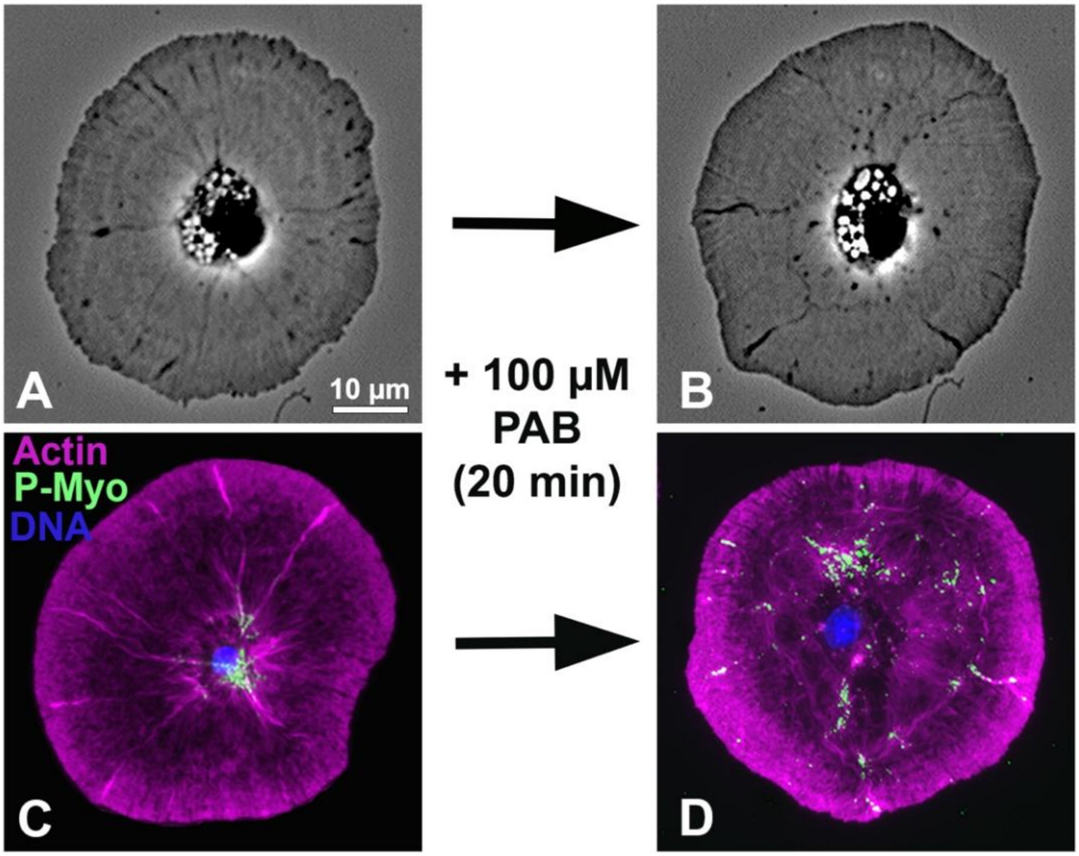

Supplementary Figure S3: PAB inhibits myosin II-dependent central centripetal flow in sea urchin coelomocytes. Live cell phase contrast video shows arrest of central flow in a +PAB cell as indicated by the loss of the radial organization of the central actin cytoskeleton (A, B). Staining for F-actin (magenta) and activated myosin II (P-Myo, green) demonstrates that PAB treatment disorders the actomyosin array that mediates the central centripetal flow in control cells (C, D). Note that peripheral centripetal flow (driven by Arp2/3-mediated actin polymerization) continues unperturbed by PAB treatment.

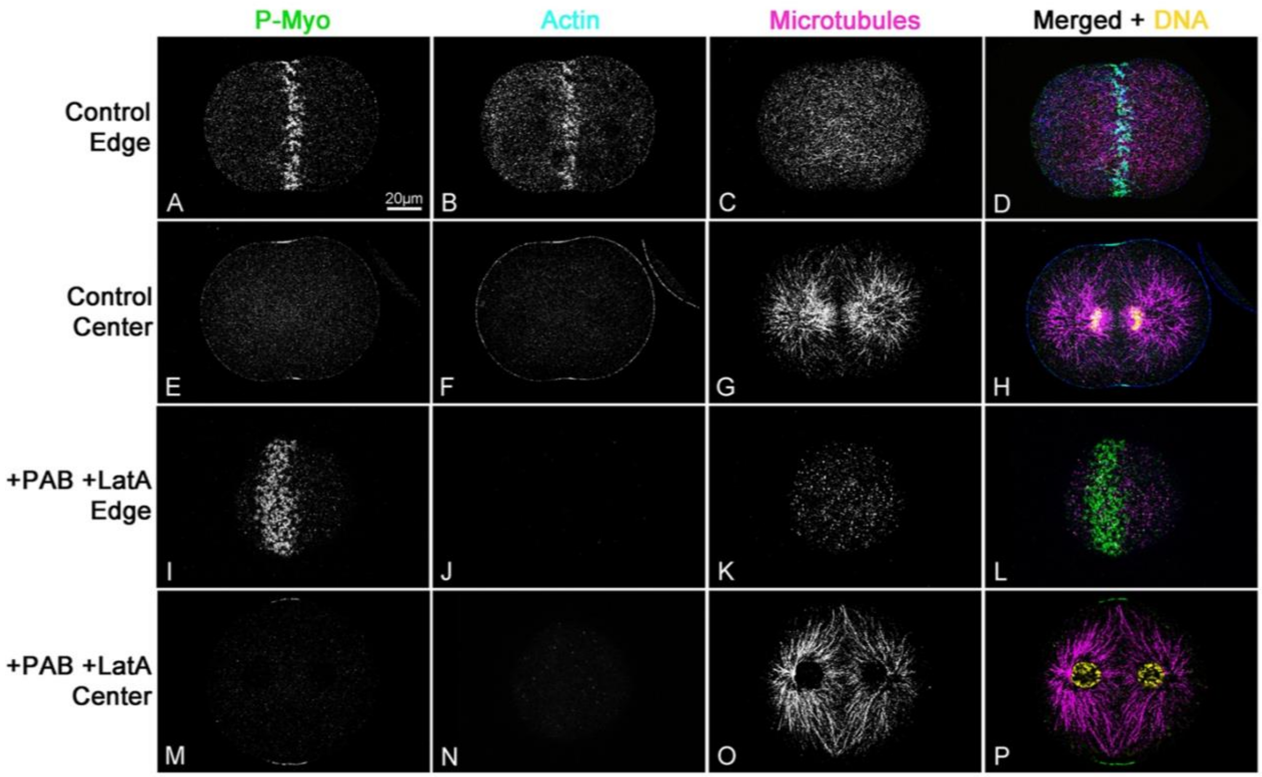

Supplementary Figure S4: The early CR template of myosin II clusters still assembles in embryos treated to remove actin filaments and inhibit myosin II. Confocal images of the edge/cortical and center/mid Z slices of a control telophase embryo shows the expected dense CR array of activated myosin II (P-Myo, A, E) and F-actin (B, F) along with microtubules (C, G). Confocal images of an embryo treated with LatA plus PAB shows a broad band of early CR-like myosin II clusters (I, M), no significant actin staining (J, N), and microtubules (K, O) that have accomplished karyokinesis in order to generate a binucleate embryo (P). Control embryo at 130 min post-fertilization and +LatA/+PAB embryos 140 min post-fertilization.

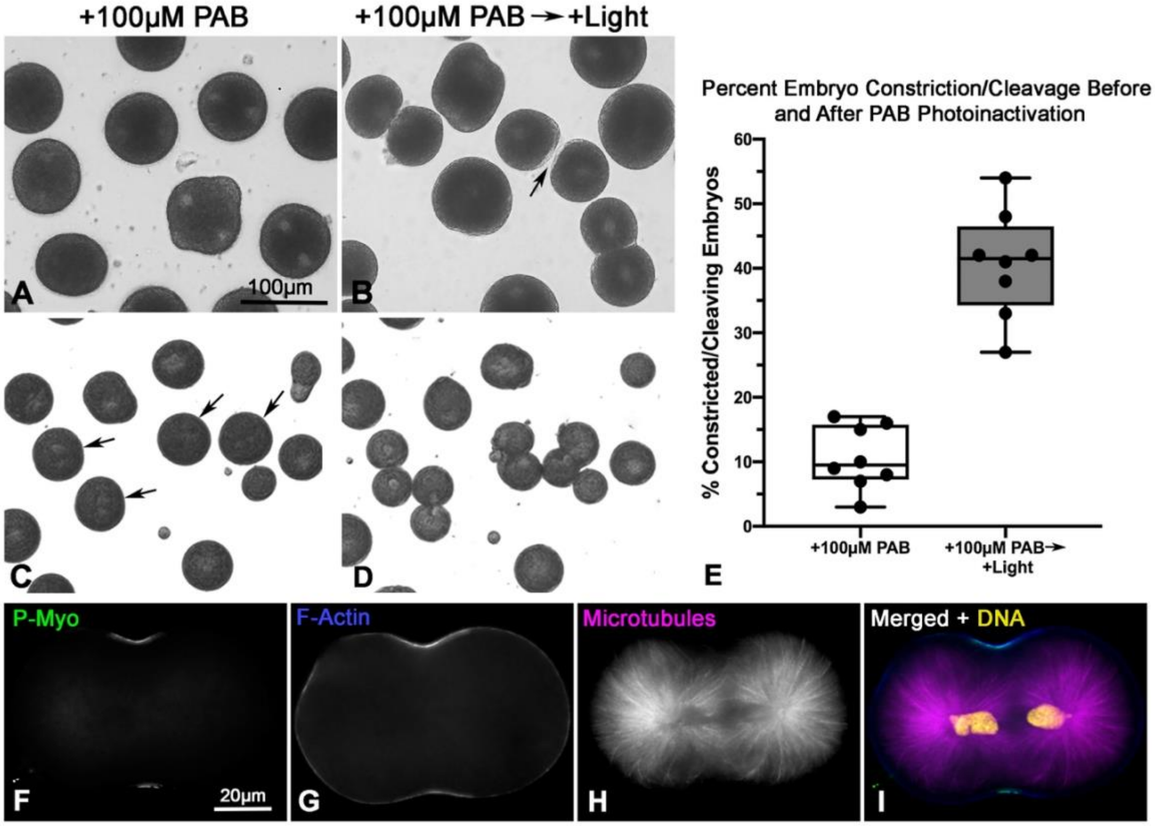

Supplementary Figure S5: The inhibition of cytokinesis in embryos treated with PAB can be reversed by photoinactivation. Live embryos treated with 100 µM PAB at the Fertilization + 160 min time point show clear inhibition of cytokinesis and are binucleated (A, C). Following 5 min of exposure to intense fiber optic white light cytokinesis resumes (B, D), with the arrow in B indicating complete abscission and the arrows in C pointing out binucleated embryos that undergo cytokinesis in panel D. Quantitative analysis of photoinactivation results from four separate experiments shows a statistically significant difference in the % embryo constrictions/cleavages before and after PAB photoinactivation (E, $p<0.0001$). Fluorescent labeling of a PAB photoinactivated embryo undergoing cytokinesis (F-I) shows the expected CR-specific concentration of activated myosin II (F, green) and F-actin (G, blue), as well as a microtubule array (H, magenta). Note that the nuclear (I, yellow) morphology of this embryo is anomalous given that division is taking place in an embryo that has already undergone karyokinesis.
